# Exploring fiberglass post numbers for enhanced fatigue resistance in molars without coronary remnants

**DOI:** 10.1590/0103-6440202405946

**Published:** 2024-09-16

**Authors:** Lais Oliveira Vazzoler, Lucas Saldanha da Rosa, Helder Callegaro Velho, Lara Dotto, Luiz Felipe Valandro, Atais Bacchi, Rafael Sarkis-Onofre, Aloisio Oro Spazzin, Rodrigo Alessandretti, Gabriel Kalil Rocha Pereira

**Affiliations:** 1 Graduate Program in Dentistry, Atitus Educação, Passo Fundo, RS, Brazil; 2 Post-Graduate Program in Oral Sciences, Center for Development of Advanced Materials, Division of Prosthodontics-Biomaterials, Federal University of Santa Maria(UFSM), Santa Maria, RS, Brazil; 3 Post-Graduate Program in Dentistry, Paulo Picanço School of Dentistry, Fortaleza, Ceará, CE, Brazil.

**Keywords:** Fatigue resistance, intraradicular posts, post and core technique, survival analysis, rehabilitation of endodontically treated teeth

## Abstract

This study aimed to assess the fatigue resistance of molars lacking a coronary remnant, using zero, one, two, or three fiberglass posts. Forty caries/crack-free human molars with coronal portions removed at the pulp chamber floor were randomly allocated into four groups (n=10). Following endodontic treatment, posts (Whitepost DC/DC.E 0.5, 1.0, FGM) were silanized (silane agent, Angelus) and cemented with a resin cement (RelyX U200, 3M ESPE). Resin composite cores (Z350, 3M ESPE) were built, and metal crowns were fabricated and cemented using the same luting system. Samples underwent cyclic fatigue testing at 45°, applying load in the occlusal surface at 10 Hz and 100 N initial load for 10,000 cycles, with 50 N increments every 10,000 cycles until failure. Fatigue failure load and cycles for failure data were recorded and subjected to survival analysis through Kaplan-Meier and Mantel-Cox post hoc tests, and Weibull analysis. Fractography patterns of failed crowns were qualitatively analyzed. The group without posts exhibited the lowest fatigue performance (p < 0.05) for both fatigue failure load and cycles to failure. Superior fatigue performance was observed in the three-post group, followed by groups with one or two posts, corroborated by the Weibull characteristic strength parameter. Weibull moduli were similar among conditions. All specimens exhibited failure involving detachment of the restorative set (posts/core/crown) with a portion of the dental remnant, without tooth fracture. Thus, when restoring mandibular molars without crown remnants, the use of fiberglass posts promotes greater fatigue resistance to oblique loads.

## Resumo

O objetivo deste estudo foi avaliar a resistência à fadiga de molares sem um remanescente coronário, utilizando zero, um, dois ou três pinos de fibra de vidro. Quarenta molares humanos livres de cárie e fissuras, com porções coronárias removidas ao nível do assoalho da câmara pulpar, foram alocados aleatoriamente em quatro grupos (n=10). Após o tratamento endodôntico, os pinos (Whitepost DC/DC.E 0.5, 1.0, FGM) foram silanizados (agente silano, Angelus) e cimentados com um cimento resinoso autoadesivo (RelyX U200, 3M ESPE). Núcleos de resina composta (Z350, 3M ESPE) foram construídos, coroas metálicas foram fabricadas e cimentadas usando o mesmo sistema de cimentação. As amostras foram submetidas a testes de fadiga cíclica a 45°, aplicando carga na superfície oclusal a 10 Hz e carga inicial de 100 N por 10.000 ciclos, com incrementos de 50 N a cada 10.000 ciclos até a ocorrência da falha. Os dados de carga de falha por fadiga e ciclos até a falha foram registrados e submetidos à análise de sobrevivência por meio de testes post hoc de Kaplan-Meier e Mantel-Cox, além de análise de Weibull. Os padrões de fratura das coroas que falharam foram analisados qualitativamente. O grupo sem pinos apresentou o desempenho mais baixo em fadiga (p < 0,05) tanto para carga de falha por fadiga quanto para ciclos até a falha. Um desempenho superior em fadiga foi observado no grupo com três pinos, seguido pelos grupos com um ou dois pinos, corroborado pelo parâmetro de resistência característica de Weibull. Os módulos de Weibull foram semelhantes entre as condições. Todas as amostras apresentaram falha envolvendo desprendimento do conjunto restaurador (pinos/núcleo/coroa) com uma porção do remanescente dentário, sem fratura do dente. Portanto, ao restaurar molares mandibulares sem remanescente coronário, o uso de pinos de fibra de vidro promove uma maior resistência à fadiga a cargas oblíquas.

## Introduction

As caries progresses and crown destruction occurs, the ultimate option to preserve tooth function is endodontic treatment. However, this treatment poses challenges, as dental elements become more prone to fracture due to extensive tissue removal ^1^. Additionally, the process results in the loss of moisture, flexibility, and reduced fracture strength, primarily attributed to endodontic access preparations ^2^ ^,^ ^3^. Despite advancements in materials and restoration techniques, root fractures persist in endodontically treated teeth ^4^. To mitigate this risk, adopting a more conservative approach during both endodontic and restorative procedures proves to be an effective measure ^4^ ^,^ ^5^ ^,^ ^6^.

Depending on the extent of tooth destruction, the crown portion may be severely compromised, posing a challenge for future restorations due to deficient retention ^5^. Clinical and radiographic assessments of the remaining coronal structure guide the determination of the need for intraradicular retainers, ensuring a favorable prognosis and extended clinical longevity ^7^ ^,^ ^8^ ^,^ ^9^. Careful consideration is essential when selecting the most appropriate retainer, given the various materials and post systems available, including cast metal posts, prefabricated posts primarily composed of fiberglass-reinforced resin, and anatomized fiberglass posts with resin composite ^5^ ^,^ ^10^.

In recent years, the most widely used intraradicular retainer in dentistry has been the fiberglass post ^11^. Its popularity stems from accessibility, cost-effectiveness, and the absence of laboratory steps. Unlike cast retainers, fiberglass posts offer favorable aesthetics and their main role is to enhance restoration retention to the remaining tooth structure ^6^. A notable advantage of fiberglass posts is their elastic modulus, which closely matches that of dentin, in contrast to cast metal posts ^5^. This property ensures a more uniform distribution of forces from occlusal loads throughout the root, reducing failures. In instances of failure, they typically occur in the restoration, which is repairable and, importantly, does not compromise the remaining root structure ^12^ ^,^ ^13^.

Some authors suggest a low need for a post in endodontically treated posterior teeth due to its preparation anatomy and its relation to the restoration ^14^ ^,^ ^15^ However, other authors argue that posterior teeth without coronary remnant and a very shallow pulp chamber may be compromised by the loss of structure, especially in situations involving translational movements ^8^ ^,^ ^16^. The nature of the applied forces in posterior teeth results in not only axial forces but also radial forces, which can be unfavorable to damaged teeth with the aforementioned characteristics. In such cases, the use of an intraradicular post may be necessary to retain the restoration.

Furthermore, another crucial factor to consider is the presence of ferrule, as highlighted in the literature emphasizing the significance of having a tooth remnant of 1.5 to 2.0 mm (ferrule) ^17^. Cases with a favorable prognosis are associated with the presence of this ferrule, compared to teeth lacking coronal structure ^18^. The optimistic outlook is attributed to the effective distribution of tensile stresses with dissipation on the external surface of the cervical third of the root. Conversely, in cases where teeth lack a ferrule, the stress distribution differs, increasing the likelihood of irreversible fracture ^19^. It is important to note that depending on the extent of tooth destruction, achieving a ferrule may not always be possible.

When deciding to use posts in posterior teeth, the number of posts is also a controversial criterion, as authors disagree on the optimal mechanical behavior of the dental element ^16^. It is noted that the literature has not reached a consensus on the need to use posts and whether a greater number of posts would improve or compromise the fatigue strength of multirooted posterior teeth ^20^. In this context, the objective of this study was to assess the fatigue resistance of molars lacking a coronary remnant, using zero, one, two, or three fiberglass posts. The hypothesis tested was that a greater number of posts increases the fatigue resistance of molars without a coronary remnant.

## Material and methods

### Experimental design

For this in vitro study, human molar teeth free of caries or cracks, were selected and their coronal portion removed at the level of the pulp chamber floor. The teeth were submitted to endodontic treatment and randomly divided into four groups (n= 10) based on the number of fiberglass posts used: without post, with one post, with two posts, and with three posts, based on a previous study ^16^. Subsequently, all teeth received a core filling after post-cementation. Full metal crowns were fabricated in a prosthetic laboratory and cemented onto the core. Following this, the specimens were subjected to a mechanical fatigue test.

### Selection and preparation of specimens

The study consisted of forty mandibular molar teeth extracted from human donors obtained through the Atitus Educação tooth bank. The research was approved by the Research Ethics Committee of Atitus Educação (CEP/Atitus Educação 4,642,281, CAAE 44923421.1.0000.5319) on April 10, 2021. Teeth were first chosen following manual assessments, including criteria such as tooth dimension (buccal-lingual and mesial-distal lengths), and root anatomy, without great curvatures, besides caries and crack-free. A second inspection was conducted through radiographic assessments, excluding any teeth with calcified canals or with increased canal curvature. and stored in distilled water at 4 °C until use. The cut was performed transversally at the cement-enamel junction using a cutting machine (Isomet 1000; Buehler Ltd, Lake Bluff, USA) equipped with a diamond disk (Buehler Ltd), operated at low speed (150 rpm) under water cooling.

### Endodontic treatment

### Root canal preparation

A #10/15 K-file (Dentsply Sirona, Charlotte, USA) was carefully inserted into the root canal until it became visible at the apical foramen, allowing for the determination of the real length of the canal. The working length (WL) was established as one millimeter shorter than the previously determined canal length.

The root canals were instrumented using mechanical files with an X-Smart Plus electric motor (Dentsply Sirona). Initially, a reciprocating file #20.07 (TDK Files/Eurodonto, Curitiba, Brazil) was employed. This file was inserted until resistance was felt and operated with three in-and-out pecking motions with light apical pressure. After the instrument removal and cleaning, a sequence of three HERO 642 rotary files with 2% taper and diameters of 0.35, 0.40, and 0.45 mm, respectively (MicroMega, Besançon, France), was used. Subsequently, the samples were prepared using mechanical files up to the #45.02 rotary file to standardize the root canals' diameters. The canals were instrumented with 2% chlorhexidine gel (Natupharma, Passo Fundo, Brazil) as a chemical auxiliary substance and rinsed with 5 mL of saline solution between each file using a needle (0.55×20 mm) placed at a 2-mm distance from the working length. Next, the root canals were irrigated with 3 ml of EDTA solution (Biodinâmica, Ibiporã, Brazil), left to act for 3 minutes, followed by a final flush with 5 ml of saline solution to remove any residue. The canals were dried with compatible paper points (Tanariman Industrial LTDA, Manacapuru, Brazil) and obturated with single gutta-percha cones (Odous de Deus, Belo Horizonte, Brazil) and Endofill sealer (Dentsply Sirona). The sealer was applied to the cone tip and inserted into the canal in a single movement. The excess part of the cone in the coronal section was removed with a heated instrument. After, new radiographs were performed to confirm the quality of the filling and to ensure the absence of visible cracks at the tooth structure. Finally, the crowns were sealed with temporary restorative material (Villevie, Joinville, Brazil), and the teeth were stored moist at 37 °C for one week.

### Core reconstruction and post-cementation

**Figure 1:**
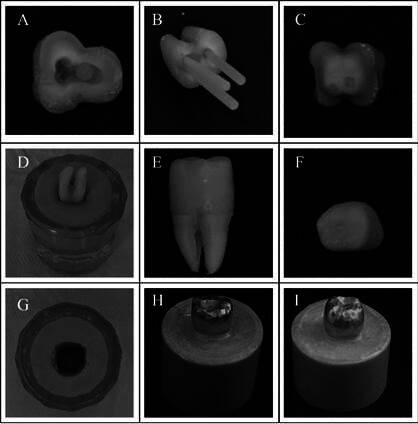
Specimen confection sequence. A-B: Selection of posts in root canals after root preparation. C-D: Master core confection and molding respectively. E-F: Constructed model crown. G: Model crown molding. H-I: Inclusion of luted samples in PVC cylinder.

The specimens were randomly (https://www.random.org) divided into four groups (n= 10) based on the number of fiberglass posts used: without post, with one post, with two posts, and with three posts. For the canals without posts, the gutta-percha was kept up to the canals’ entrances. For the canals receiving posts, the preparation involved removing 2/3 of the gutta-percha using Gates Glidden burs #2 and #3 (Dentsply Sirona) and a wide bur #1 #2 (Dentsply Sirona), while maintaining at least 3 mm of the apical seal. In the case of using one post, the distal canal was prepared. For two posts, the distal and mesiolingual canals were prepared. If three posts were used, the mesiobuccal, mesiolingual, and distal canals were prepared. A follow-up radiograph confirmed the canal preparation, and the posts were tested. Finally, the most suitable posts for the canal sizes were selected (Figure 1).

After cleaning with 70% alcohol, a silane agent (Angelus, Londrina, Brazil) was applied to the fiberglass posts (Whitepost DC 0.5, 1.0, and Whitepost DC.E 0.5, 1.0, FGM) and left for a 1-minute reaction time. The root canal was irrigated with saline solution and dried with paper points (Tanariman Industrial LTDA), then received dual-cure self-adhesive resin cement (RelyX U200, 3M ESPE, St. Paul, USA) manipulated according to the manufacturer’s instructions. The cement was inserted into the root canal using a K #15 endodontic file (Dentsply Sirona). The posts were inserted into the root canals with manual pressure, and excess cement was removed with a micro brush (KG Sorensen, Serra, Brazil). Subsequently, the cement was photoactivated (Valo, Ultradent Products, Inc., South Jordan, USA) for 40 seconds through the post and recommended by the manufacturer.

After installing the posts, a resin composite master core (Z350, 3M ESPE) was incrementally built. Initially, the height was standardized at 7.0 mm, and the circumferential wear was set at 1.0 mm. An impression of the preparation was made using silicone (Express XT, 3M ESPE). This impression served as a template to standardize all preparations (Figure 1). Subsequently, the template was cut vertically, and a thin layer of resin composite was placed inside the template and light-activated for 20 seconds, creating a central matrix of composite resin. This matrix was filled with resin composite and, above the remaining post, coated with resin composite and light-activated for 20 seconds. The other half of the preparation followed the characteristics of the template.

### Fabrication of crowns and luting procedure

A model crown with a height of 9 mm and a thickness of 1 mm was created to standardize the size and shape of the prosthetic restoration. A concavity was designed on the crown's occlusal surface to prevent slipping in the testing machine and served as a standardized point of force application on all specimens. A model impression of the crown was made using additional silicone (Express XT, 3M ESPE) to create a template matrix for uniform acrylic crowns (Figure 1). Self-curing acrylic resin (Dencrilay, VIPI, Pirassununga, Brazil) was dispensed into this matrix and applied to the preparation. After polymerization, the matrix was removed, and the crown was relined. Finally, the acrylic crowns were pressed with nickel-chromium alloy using the lost wax technique.

For the luting procedure, the inner surface of the crown was air-abraded using an oscillatory movement with aluminum oxide 90 µm (Bio-Art Equipamentos Odontológicos Ltda., São Carlos, SP, Brazil) for 15 s at 2.8 bar of pressure and 10 mm of distance. Subsequently, the cores and crowns were cleaned with cotton wool soaked in 70% alcohol and air-dried.

All metallic crowns were luted onto the cores using dual self-adhesive resin cement (RelyX U200, 3M ESPE). The resin cement was manipulated following the manufacturer’s instructions. Subsequently, the mixture was inserted inside the crown, and 7.5 N was applied until its final position. Finally, excess material was removed with a microbrush (KG Sorensen) and 6 minutes waited for the cement cure.

The specimens were then embedded in polyvinyl chloride (PVC) cylinders (25 mm in diameter and 20 mm in height) with acrylic resin (Vipiflash; VIPI), leaving 3 mm of the cervical portion of the exposed root without periodontal ligament ^21^.

### Fatigue resistance

Fatigue testing was executed with a cyclic fatigue methodology ^22^. An electric testing machine (ElectroPuls E3000; Instron) was used with each crown positioned at a 45-degree inclination. The assemblies were submerged in water, and a 6 mm diameter stainless-steel sphere piston applied the load at the center of the crowns. To enhance contact and prevent damage, a 110 μm adhesive tape was placed between the piston and the crown. Cyclic loads were applied at 10 Hz, starting from 100 N, followed by sequential 50 N increments every 10,000 cycles until failure. Specimens were inspected after each testing step or if the machine detected excessive displacement. Failure data, including fatigue failure load (FFL) and cycles for failure (CFF), were recorded for statistical analysis. After failure, specimens were examined under a stereomicroscope (Discovery V20, Carl Zeiss, Gottingen, Germany) with a 10× magnification lens (Achromat S 0.5× FWD 151mm, Carl Zeiss, Gottingen, Germany) to determine the failure pattern.

### Statistical analysis

A post hoc power analysis was conducted to determine if the sample size was adequate to support the found results (G*Power; Heinrich-Heine-Universitit, Diisseldorf, Germany). Fatigue failure load (FFL) and cycles for failure (CFF) underwent survival analysis using Kaplan-Meier and Mantel-Cox post hoc tests with IBM SPSS Statistics v21 (IBM Corp) software (α= 0.05). Survival rates were calculated for both parameters at different testing steps. Weibull analysis was performed (Super-SMITH; Fulton Findings, California, USA) to assess structural reliability. It was obtained Weibull moduli and their respective 95% confidence intervals for FFL and CFF. The characteristic strength parameter for both outcomes (where 63.2% of specimens presented failure for each condition) was determined. Statistical differences in Weibull moduli were assessed using the maximum likelihood approach, with overlapping confidence intervals indicating statistical similarity and non-overlapping ones indicating statistical differences. Fractography patterns of failed crowns were qualitatively analyzed based on a previous study ^23^. The failures were considered as favorable when occurring in the cervical third of the root or unfavorable when involving the middle and apical thirds of the root.

## Results

Based on the obtained data, the accomplished sample power was 1.00. The Kaplan-Meier and Mantel-Cox post-hoc tests for survival analysis (Table 1) indicate a statistically significant impact of the number of posts on the fatigue performance of restored teeth. Specifically, the group without posts exhibited the poorest fatigue performance (p < 0.05) for both FFL and CFF. The three-post group showed the highest fatigue performance (FFL and CFF), with the second-best performance observed in groups with one or two posts (Table 1). Characteristic parameters of Weibull analysis (Table 1) for FFL and CFF support these findings (Three posts > Two posts = One post > Without post). There were no significant differences in Weibull modulus for FFL and CFF among groups (Table 1).

In terms of survival rates (Table 2), it is evident that the group without posts exhibited an elevated risk of premature failure compared to the other groups. It initiated failure at the 100 N/10,000 cycles step, with all specimens failing before the 250 N/40,000 cycles step. In contrast, the other groups had less than a 50% failure rate at this step (250 N/40,000). Additionally, the group with three posts had a 44% chance of surpassing the 500 N/90,000 cycles step, while all other groups had already failed (0% survival chance at this step).

**Figure 2:**
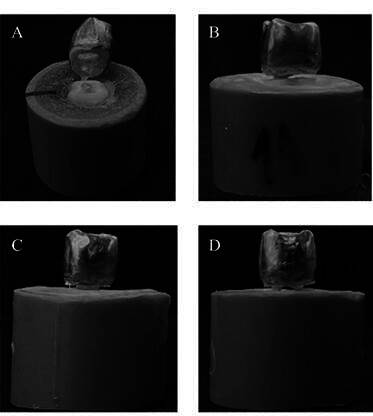
Representative illustrative figures of each group that failed the fatigue test. Groups: A - Without post, B - One post, C - Two posts, and D - Three posts. It is possible to observe the debonding between post(s) and tooth by the gap or total detachment of the crown in the group without posts.

Regarding failure patterns (Figure 2), all fractures were considered favorable, exhibiting failure involving detachment of the restorative set (posts/core/crown) along with a portion of the dental remnant, without actual tooth fracture.

**Table 1:** Results (Mean and standard deviation) for fatigue failure load (FFL), number of cycles until failure (CFF), Characteristic parameter, and Weibull modulus (Mean and 95% confidence intervals).

Groups	FFL			CFF		
	Kaplan-Meier analysis and Mantel-cox post-hoc tests*	Weibull analysis**		Kaplan-Meier analysis and Mantel-cox post-hoc tests*	Weibull analysis**	
	Mean (Standard Deviation)	Characteristic parameter (95% Confidence interval)	Weibull modulus (95% Confidence interval)	Mean (Standard Deviation)	Characteristic parameter (95% Confidence interval)	Weibull modulus (95% Confidence interval)
Without post	166.67 ^C^ (16.67)	184.30 ^C^ (150.50 - 222.80)	3.96 ^A^ (2.21 - 6.28)	23,334 ^C^ (3,334)	26,302 ^C^ (19,575 - 34,682)	2.73 ^A^ (1.50 - 4.37)
One post	333.33 ^B^ (31.18)	365.30 ^B^ (310.10 - 426.40)	5.01 ^A^ (2.61 - 8.59)	56,667 ^B^ (6,237)	62,700 ^B^ (51,288 - 75,872)	4.08 ^A^ (2.12 - 6.99)
Two posts	311.11 ^B^ (33.10)	345.90 ^B^ (274.70 - 429.30)	3.44 ^A^ (1.98 - 5.29)	52,223 ^B^ (6,621)	58,364 ^B^ (44,728 - 75,557)	2.93 ^A^ (1.68 - 4.52)
Three posts	494.44 ^A^ (55.56)	549.60 ^A^ (439.40 - 678.40)	3.60 ^A^ (1.97 - 5.76)	88,889 ^A^ (11,112)	99,341 ^A^ (77,185 -126,020)	3.19 ^A^ (1.74 - 5.13)

* Different letters on these columns indicate statistical differences between evaluated conditions depicted by Kaplan-Meier and Mantel-Cox post-hoc tests (α = 0.05).

** Different letters on these columns indicate statistical differences between evaluated conditions depicted by Weibull Analysis, based on the absence of overlap of 95% confidence intervals (maximum likelihood estimation).

**Table 2:** Survival rates, obtained at the Kaplan-Meier survival test, indicate the probability of the specimens of such condition exceeding the respective fatigue failure load (FFL) and number of cycles until failure (CFF) number step without failure and its respective standard error values.

Groups	FFL (N) / CFF (Count)													
	100/ 10,000	150/ 20,000	200/ 30,000	250/ 40,000	300/ 50,000	350/ 60,000	400/ 70,000	450/ 80,000	500/ 90,000	550/ 100,000	600/ 110,000	650/ 120,000	700/ 130,000	750/ 140,000
Without post	0.78 (0.14)	0.44 (0.17)	0.11 (0.11)	0.0	-	-	-	-	-	-	-	-	-	-
One post	1.0	1.0	1.0	0.67 (0.16)	0.67 (0.16)	0.56 (0.17)	0.0	-	-	-	-	-	-	-
Two posts	1.0	1.0	0.89 (0.11)	0.56 (0.17)	0.22 (0.14)	0.22 (0.14)	0.22 (0.14)	0.11 (0.11)	0.0	-	-	-	-	-
Three posts	1.0	1.0	0.89 (0.11)	0.89 (0.11)	0.89 (0.11)	0.78 (0.14)	0.67 (0.16)	0.56 (0.17)	0.44 (0.17)	0.33 (0.16)	0.22 (0.14)	0.11 (0.11)	0.11 (0.11)	0.0

* The sign ‘-’ indicates the absence of a specimen of such condition being tested at this respective step.

## Discussion

The present study investigated whether molars with no coronal remnants with an increasing number of fiberglass posts would exhibit better fatigue resistance. Results showed these teeth had higher survival rates when fitted with three posts compared to those with one or two posts, which demonstrated similar performance. Overall, all conditions with posts showed better fatigue performance than teeth without posts. In general, the number of posts in molars without crown remnants influenced fatigue strength, with a higher number of posts improving mechanical performance, thus the tested hypothesis could not be rejected.

The literature presents conflicting views on the number and size of posts. Barcelos et al. (2017) ^16^ found that using a single fiberglass post provided higher fracture resistance and better stress distribution (lower strain and stress concentration) than using two posts. They also saw more reparable failure modes in comparison with the group without posts ^16^. However, the present study contradicts those findings, showing better results in the group with three posts, which had a 44% chance of exceeding 500 N/90,000 cycles, while the other groups failed before reaching that point. Besides, in the present study, all failures were detachment of the restorative set without fracture of the tooth remnant. Barcelos et al. justified their results using the Saint-Venant principle, suggesting that stress distribution becomes uniform at a certain distance from the point of application. In their study, the presence of two fiberglass posts led to stress concentration close to the loading area, reducing fracture strength due to high-stress concentration near the load application point.

A crucial difference from the aforementioned study is the simulation of oblique forces instead of axial forces. Oblique forces occur clinically when the mandible makes lateral movements. In the axial direction, the force happens vertically, producing a more significant wedge effect ^13^. Therefore, exploring the simulation of excursive movements, which is not often explored in vitro research, is considered a relevant approach and one of the main novelties of the present data. Given the nature of such movements, it is assumed that the use of additional posts in molars promotes better stress distribution ^24^. Furthermore, using fiberglass posts offers the advantage of bonding interaction with the restorative material, the cement used, and the remaining tooth structure. This interaction creates a "monoblock" design that dissipates stresses produced by occlusal loads ^25^ ^,^ ^26^.

In the premolar context, the use of multiple posts is shown as a positive technique to reinforce the dental structures. In a study conducted by Spicciarelli et al. ^27^, using one or two posts in premolars increased the axial load to fracture resistance compared to not using posts. It is interesting to note that when the teeth presented all residual walls, one single post presented increased fracture resistance than two posts. Controversially, in the present study, there were no statistical differences between the groups with one or two posts. In another study conducted by Fráter et al. ^28^, multiple conventional fiberglass posts presented increased oblique load to fracture resistance compared to a single post. This finding is by the present research and the load incidence could contribute to the observed results.

The group with three posts demonstrated the best fatigue performance, suggesting that a greater number of conduits allowed for better load distribution along the restorative set and increased survival. The literature shows that preparing the space for post-insertion weakens the structure and increases fracture probability ^29^. Based on this, post-insertion should not be at the expense of excessive root dentin removal ^29^ ^,^ ^30^, as the remaining tooth structure plays a crucial role in terms of strength, and fracture resistance ^30^ ^,^ ^31^. However, when using an appropriate technique, the insertion of three posts may not necessarily involve excessive dentin removal and weakening of the tooth. Proper instrumentation during endodontic treatment can contribute to the modeling of the root canal, and selecting an appropriate post system ensures that canal preparation does not lead to excessive dentin removal ^32^. The literature also supports the possibility of using accessory posts, where a "master" canal is prepared following biomechanical principles ^30^,^33^, and additional canals are prepared with smaller calibers and lengths, as mentioned by Mayya et al. (2020) ^31^, preserving root dentin while retaining the restoration effectively.

The longevity and clinical success of post-retained restorations are influenced by factors such as selecting the correct post length and diameter, and the quantity and quality of remaining coronary dentin ^32^ ^,^ ^34^. Evidence suggests that having a ferrule of at least 1.5 mm in height can act as a lever arm, impeding the progression of fracture and improving restoration longevity ^5^ ^,^ ^17^ ^,^ ^18^. However, there are conflicting findings; a prospective clinical trial reported similar survival probabilities for restorations with or without a ferrule ^12^. In this study, an unfavorable scenario was created by including teeth without circumferential dentin, thereby preventing the ferrule effect.

In this work, specimens underwent oblique loading to simulate mandibular excursive movements ^5^. While this simulation induces increased shear force, it's noteworthy that fiber posts typically consist of longitudinal fibers in an epoxy resin matrix, capable of withstanding high tensile stresses but more susceptible to failure under shear stresses ^35^. Under such forces, failure occurs at the luting interface, allowing movement of the restorative set ^35^. This aligns with our study's findings, where a 100% displacement of the posts/core/crown set was observed.

The present study used different post diameters depending on the tooth and also used a strict variety of small post diameters to fit the used teeth. However, a study by Bacchi et al. (2013) ^33^ revealed that increased post diameter did not contribute to enhanced fracture resistance, advocating for the use of smaller-diameter posts to preserve root dentin. Further research in this area is encouraged, especially considering finite element analysis to better understand the stress distribution in such scenarios. Additionally, studies have suggested that simulating the periodontal ligament has no impact on fracture or bond strength between fiberglass posts and cores. Consequently, this study did not simulate the periodontal ligament ^21^.

Overall, a greater number of fiberglass posts may improve the fatigue mechanical strength of posterior teeth with limited coronary remnants or shallow pulp chambers. Despite that, there are study limitations that should be pondered. In the present investigation, despite using an oblique load (45-degree inclination), it may not completely mimic the complex movement involved during function. Furthermore, the use of ceramic crowns can lead to different types of failures, such as restoration fractures. Also, no aging protocols were employed, restricting the extrapolation of the present findings. Future studies would benefit from finite element analysis to better comprehend the stress distribution over the restored set. It is worth mentioning that despite the findings from the present study, clinical trials are essential to validate the results of this laboratory study and base a clinical protocol.

## Conclusion

For the restoration of mandibular molars without crown remnants, the use of fiberglass posts enhances fatigue resistance during oblique loads.
